# Multi-subunit collaboration enables Smc5/6 to function as a composite SUMO E3 complex

**DOI:** 10.21203/rs.3.rs-9557402/v1

**Published:** 2026-04-30

**Authors:** Xiaoyu Xue, Jiayi Fan, Shibai Li, Sofya Ignatyeva, Patricia Gallegos-Elias, Heather McEntire-Benitez, Xinji Zhu, Tanu Kar, John Epps, Lillian Eliaz, Kiana Holland, Andrea Romero, Xiaolan Zhao

**Affiliations:** 1 Department of Chemistry and Biochemistry, Texas State University, San Marcos, Texas, USA; 2 Materials Science, Engineering, and Commercialization Program, Texas State University, San Marcos, Texas, USA; 3 Integrated Molecular and Biophysical Chemistry Program, Texas State University, San Marcos, Texas, USA; 4 Molecular Biology Program, Memorial Sloan Kettering Cancer Center

**Keywords:** Smc5/6, SUMO E3, DNA binding, ATPase, Sgs1

## Abstract

SUMO E3 enzymes control the efficiency and specificity of protein SUMOylation, providing regulatory means for many cellular processes. While most SUMO E3s fulfill their roles as single proteins, the conserved Nse2 E3 is an obligatory subunit of the genome-protecting complex Smc5/6. How the Smc5/6 complex functions in SUMOylation and the roles of its non-SUMO E3 subunits in this process remain to be elucidated. Here we examine the budding yeast Smc5/6 in SUMOylation reactions and in cellular SUMOylation assays. Biochemical data show that DNA stimulates Smc5/6’s E3 activity via fostering enzyme and substrate proximity. Mutational analyses reveal that four non-SUMO E3 subunits utilize their DNA binding abilities to support this stimulation. Moreover, ATP binding by SMC subunits favors SUMOylation by enhancing Smc5/6 association with DNA and chromatin and by enabling conformational changes. Our findings thus provide evidence for a specialized DNA- and ATP-stimulated composite SUMO E3 complex that uses inter-subunit collaboration to achieve efficient SUMOylation in genome regulation.

## INTRODUCTION

Protein modification by the SUMO modifiers, which are approximately one-hundred amino acid long proteins, provides versatile regulations of many cellular processes. SUMOylation can alter numerous properties of substrates, such as their interactions with other proteins and with DNA or their cellular localizations, leading to a range of changes in cellular pathways^1,2^. The covalent linkage of a SUMO modifier to a lysine residue of a substrate is mediated by the sequential actions of a trio of SUMO enzymes. The SUMO E1 is first conjugated to SUMO at the expense of ATP hydrolysis. SUMO E1 then passes SUMO to the SUMO E2, which collaborates with a SUMO E3 to transfer SUMO to the substrate^3^. Eukaryotic cells possess a single pair of SUMO E1 and E2, but multiple E3s that are important for achieving SUMOylation specificity and efficiency. Most SUMO E3s are single protein enzymes, such as the Siz proteins in budding yeast and their ortholog PIAS proteins in humans^4^. These E3s can simultaneously interact with the SUMO E2 and the substrate and support a productive conformation for SUMO transfer^5–7^.

Distinct from single-protein SUMO E3s, the Nse2 SUMO E3 is an obligatory subunit of Smc5/6, a genome maintenance complex with roles in recombinational repair, DNA replication, and other processes^8–13^. Besides Nse2, Smc5/6 also contains seven non-SUMO E3 subunits. For the budding yeast Smc5/6 complex examined in this work, its subunits include the highly conserved Smc5, Smc6, the Nse1-3-4 subcomplex composed of Nse1, Nse3, and Nse4, and the less conserved Nse5-6 subcomplex composed of Nse5 and Nse6, which is homologous to SLF1-SLF2 or SIMC1-SLF2 in humans^12–17^. For simplicity, we refer to the budding yeast Smc5/6 complex containing all eight subunits as holo-Smc5/6 and the one lacking Nse5-6 as core-Smc5/6 hereafter.

How a multi-subunit Smc5/6 complex functions in SUMOylation and what the roles of its non-SUMO E3 subunits (Smc5, Smc6, Nse1, Nse3-6) are in this process remain to be elucidated. *In vitro* analyses of Smc5/6 and its subcomplexes have shown that some non-SUMO E3 (non-E3) subunits have ATP and DNA binding abilities^12,13^. The Smc5 and Smc6 head domains can sandwich two ATP molecules between them to enable their head domain association, while ATP hydrolysis leads to head domain dissociation^18–23^. Importantly, this alteration induces large conformational changes to the entire complex. ATP-binding enables an O-shaped conformation wherein the two long SMC coiled coil arm regions emanating from the head regions separate from each other, whereas ATP hydrolysis drives the complex into an I-shaped configuration in which the arm regions zip up^18–24^. Biochemical and single molecule studies further show that ATP-bound Smc5/6 exhibits a greater dsDNA binding capacity, suggesting that ATP binding can influence DNA association in addition to affecting the complex conformations^25–30^.

**FIGURE 1.**
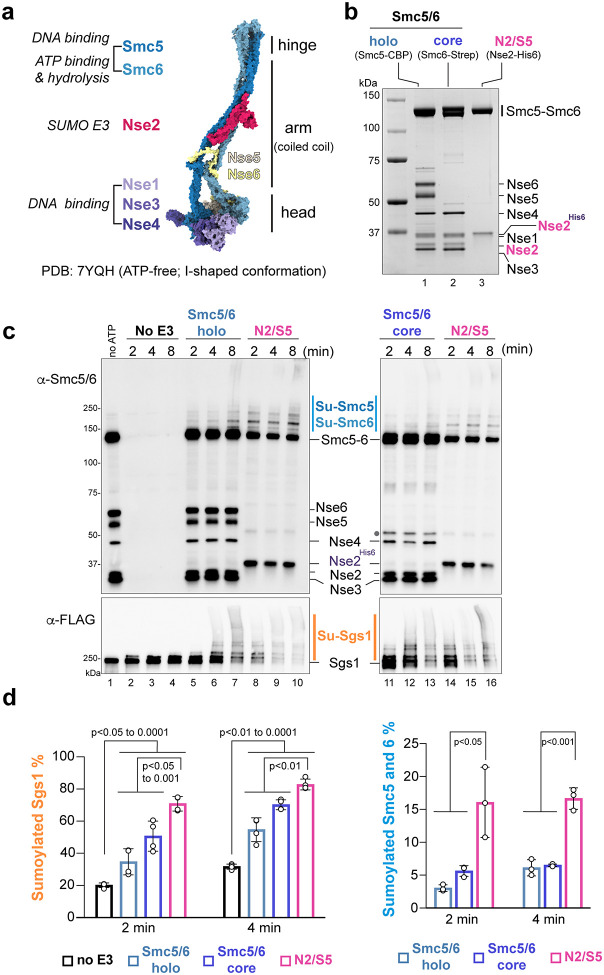
Smc5/6 supports SUMOylation of Smc5, Smc6 and Sgs1 *in vitro*. **a)** Overview of the budding yeast Smc5/6 structure, subunits, and activities. A cryo-EM structure of holo-Smc5/6 in this ATP-free, I-shaped configuration (PDB: 7YQH)^23^ is shown with eight subunits and structural elements labeled. Main activities are also indicated. **b)** Purified holo-Smc5/6, core-Smc5/6, and the Nse2-Smc5 dimer (N2-S5) complexes. The subunit fused with a protein tag for affinity purification is indicated. The complexes were analyzed by SDS-PAGE and a Coomassie blue stained gel picture is shown, with each subunit labeled. Smc5 and Smc6 have similar molecular weights and thus migrate similarly on the gel, and their bands are marked as Smc5-Smc6, whereas His6-tagged Nse2 migrated slower than untagged Nse2. **c)** Holo- or core-Smc5/6 supports *in vitro* SUMOylation. SUMOylation reactions contained SUMO, the SUMO E1, the SUMO E2, Flag-tagged Sgs1, and Top3-Rmi1. SUMO E3 was added in the form of core-Smc5/6, holo-Smc5/6 or the Nse2-Smc5 (N2-S5) dimer. Control reactions omitted ATP to prevent SUMO E1 activation. Samples were collected at the indicated time points after ATP was added and were examined by immunoblotting. A pan-Smc5/6 antibody (-Smc5/6) detected all subunits except Nse1. SUMOylated forms of Smc5 and Smc6, which migrated to similar positions on gels due to closely matched molecular weights, are marked as Su-Smc5 and Su-Smc6. SUMOylated forms of Sgs1 (Su-Sgs1) include its mono-SUMOylated as well as its poly- or multi-SUMOylated form that are difficult to separate by SDS-PAGE due to the large size of Sgs1. Unspecific bands in lanes 11–13 are marked by dots. **d)** Percentages of SUMOylated Smc5 and Smc6 or Sgs1. Means and standard deviations (SD) are shown, with p values indicated. For each condition, four and three independent experiments were performed to examine Sgs1 and Smc5/6 SUMOylation, respectively.

In both ATP-bound and ATP-free conformations, holo- and core-Smc5/6 adopt elongated shapes approximately 46 nm in length^18–24,26,27,31^. The ATP-free form of the yeast holo-Smc5/6 has been visualized at high resolution in its entire length by cryo-EM studies (Fig. 1a)^23^. In this I-shaped conformation, the head domains of Smc5 and Smc6 and the DNA-binding subcomplex Nse1-3-4 are in proximity, generating a structural module with both ATP- and DNA-binding activities (Fig. 1a). Spatially distantly located from this module is the Nse2 SUMO E3, which binds to the arm regions of Smc5 and Smc6^23,32,33^. Earlier studies examined the Nse2-Smc5 dimer in SUMOylation and found that ssDNA is superior to dsDNA in enhancing SUMOylation activity, with Smc5-arm mediating ssDNA binding^34^. However, recent studies of holo- and core-Smc5/6 complexes found avid dsDNA association and dsDNA binding residues were mapped to the head domains of Smc5 and 6 and on Nse3 and Nse4^21^. The different DNA binding sites and behaviors observed for Nse2-Smc5 dimer versus Smc5/6 complexes, as well as the observation that ATP binding requires both Smc5 and Smc6, suggest that examining the complete complex, rather than just the Nse2-Smc5 dimer, is required to gain mechanistic insights into how the E3 activity of Smc5/6 can be regulated by DNA, ATP, and the roles of its various subunits in this regulation.

Here we examined *in vitro* SUMOylation activities of the purified budding yeast core- and holo-Smc5/6 complexes and conducted complementary cellular studies. *In vitro* results show that dsDNA or dsDNA-containing structures mimicking DNA repair intermediates are superior to ssDNA in stimulating Smc5/6 SUMOylation activities. Biochemical data further suggest that the positive effect of DNA on SUMOylation can be explained by promoting substrate and enzyme proximity. Moreover, both *in vitro* and cellular studies demonstrate that several non-SUMO E3 subunits utilize their dsDNA-binding activities to promote SUMOylation. The *in vitro* and *in vivo* investigations also converge to show that ATP binding by two SMC subunits stimulates SUMOylation via enhancing complex association with DNA as well as by supporting the O-shaped conformation that is favorable for the SUMO E3 activity. Collectively, our data suggest that Smc5/6 is a composite SUMO E3 that utilizes collaboration from multiple subunits to enhance SUMOylation efficiency and specificity for chromatin-associated substrates.

## RESULTS

### *In vitro* assays to assess Smc5/6-based SUMOylation of physiological substrates

To understand how Smc5/6 complexes function in SUMOylation, we performed *in vitro* SUMOylation reactions using purified budding yeast SUMO, SUMO E1, SUMO E2, and core- or holo-Smc5/6 (Supplementary Fig. 1a; Fig. 1b). The Nse2-Smc5 dimer was purified and used for comparison (Fig. 1b). We examined three known Nse2 substrates, including the DNA repair helicase Sgs1 and Smc5 and Smc6 themselves. Previous studies have established that Nse2 E3 mutants reduce the SUMOylation levels of the three proteins in cells and that Nse2-Smc5 dimer can SUMOylate these proteins *in vitro*^9,35–37^. These proteins are also conserved substrates between yeast and humans and their SUMOylation regulates genome stability^10,35–40^. We reasoned that examining these substrates could offer insights into SUMOylation of substrates outside the complex as well as the self-SUMOylation within the E3-complex. Purified Flag-tagged Sgs1 along with its partner complex Top3-Rmi1 were included in the reactions to better mimic physiological states (Supplementary Fig. 1a). To gain a dynamic view of the reactions, products were examined at three timepoints. Immunoblotting using a pan-Smc5/6 antibody detected all subunits of Smc5/6 except Nse1, while an -Flag antibody was used to detect Flag-Sgs1. Negative control reactions omitted ATP to prevent SUMO E1 activation^41^.

As seen previously, a low level of Sgs1 mono-SUMOylation was detected without E3, reflecting basal SUMO conjugation by the E2 (Fig. 1c, lanes 1–4)^42,43^. Compared with no-E3 reactions, much enhanced Sgs1 SUMOylation was seen in reactions containing E3 proteins. As reported, upshifted Sgs1 bands representing poly- and multi-SUMOylated Sgs1 were seen when Nse2-Smc5 was included (Fig. 1c, lanes 8–10, 14–16)^42,43^. These Sgs1 SUMOylation forms (Su-Sgs1) were also detected when holo-Smc5/6 (Fig 1c, lanes 5–7) or core-Smc5/6 (Fig 1c, lanes 11–13) were included, suggesting that these Smc5/6 complexes can act as SUMO E3s *in vitro*.

As Smc5 and Smc6 are similar in size (126 and 128 kDa), their SUMOylation forms are difficult to separate on gels. We first verified that both proteins were SUMOylated, since upshifted bands were detected for CBP-tagged Smc5 within holo-Smc5/6 (Supplementary Fig. 1b, lanes 2–4 vs. lane 1) and for Strep II tagged-Smc6 within core-Smc5/6 (Supplementary Fig. 1c, lanes 2–4 vs. lane 1). Upshifted bands above unmodified Smc5 and Smc6 were observed when holo-Smc5/6 (Fig. 1c, lanes 5–7) or core-Smc5/6 (Fig. 1c, lanes 11–13) were used in reactions and were absent in no-ATP reactions (Fig. 1c, lane 1). The observations suggest that these upshifted bands present a combination of Smc5 and Smc6 SUMOylated forms, which are referred to as SUMOylated Smc5-Smc6 hereafter for simplicity.

To gain a quantitative view of the SUMOylation efficiencies for the three forms of E3, we calculated the percentages of SUMOylated Sgs1 or Smc5-Smc6 relative to their unmodified forms at two time points of the reactions. All three E3 forms stimulated Sgs1 SUMOylation compared with the no-E3 reactions, with Nse2-Smc5 generating better yields (Fig. 1d, left). The levels of SUMOylated Smc5-Smc6 produced by holo- or core-Smc5/6 were also lower than those generated by Nse2-Smc5 (Fig. 1d, right). Our assessments thus suggest that Nse2-Smc5 exhibits an overall better activity than Smc5/6 complexes in the absence of other co-factors.

### dsDNA is superior to ssDNA in stimulating Smc5/6-mediated SUMOylation

**FIGURE 2.**
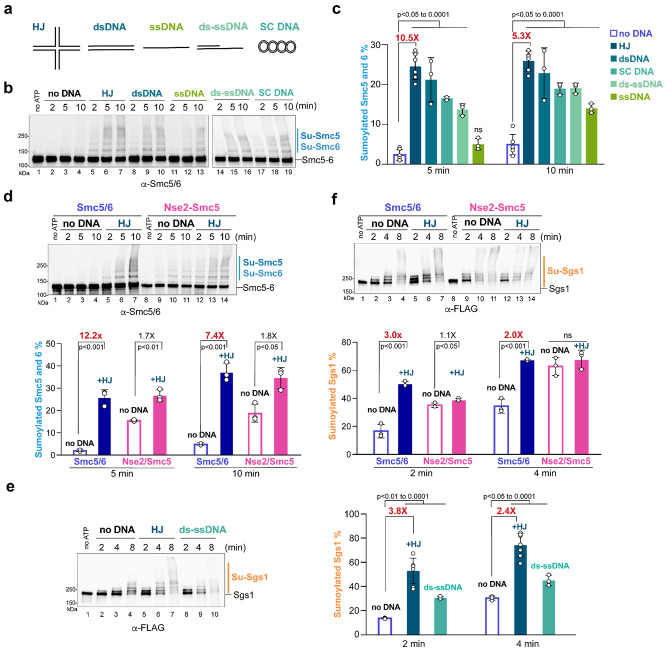
Multiple forms of DNA stimulate SUMOylation by Smc5/6. **a)** Diagrams of the five types of DNA examined in SUMOylation reactions. **b)** Five forms of DNA stimulate Smc5 and Smc6 SUMOylation. Reactions were performed as in Figure 1c, except that the core-Smc5/6 E3 was added at 25 nM and the indicated types of DNA were added at 12.8 μM nucleotide concentration. **c)** Percentages of SUMOylated Smc5-Smc6. Reactions shown in panel b and at least three independent reactions per condition were quantified and results are plotted as in Figure 1d with means, SDs, and p-values calculated from Welch’s t-test indicated (n=6 for no DNA and HJ; n=3 for other reactions). SUMOylated Smc5-Smc6 levels are not statistically significant (n.s.) between no-DNA and ssDNA samples at 5 min. Different yields of SUMOylated Smc5-Smc6 between no-DNA and HJ-DNA containing reactions are indicated as fold-changes. **d)** Comparison of Smc5-Smc6 SUMOylation between Smc5/6 and the Nse2-Smc5 E3 forms. SUMOylation of Smc5 and Smc6 was examined with or without HJ DNA using either core-Smc5/6 or Nse2-Smc5 as the SUMO E3. (Top) An example of immunoblots detecting the SUMOylated forms of Smc5 and Smc6 (Su-Smc5, Su-Smc6). (Bottom) Quantification of SUMOylated Smc5 and Smc6 in three independent SUMOylation reactions (bottom), with means, SDs, p-values, and fold changes between reactions containing no DNA and HJ-DNA indicated. **e)** HJ or ds-ssDNA promote Sgs1 SUMOylation by Smc5/6. (Left) Reactions were performed as in panel b, except that 100 mM KCl was used to reduce poly- and multi-SUMOylation of Sgs1, which appeared as a smear of bands on immunoblots. (Right) Percentages of SUMOylated Sgs1 (Su-Sgs1) are plotted. Reactions shown in panel e and at least three independent reactions per condition were quantified and plotted as in panel c with means, SDs, and p-values calculated from Welch’s t-test indicated (n=7 for HJ; n=3 for other reactions). Fold-changes indicate different yields of SUMOylated Sgs1 between no-DNA and HJ-DNA containing reactions. **f)** Comparison of Sgs1 SUMOylation enabled by Smc5/6 or the Nse2-Smc5 E3 form. Sgs1 SUMOylation was examined and quantified using either core-Smc5/6 or Nse2-Smc5 as the SUMO E3. Experiments were conducted, and data are presented as in panel d, except that the SUMOylated forms of Sgs1 were examined and quantified (n=3).

Nse2 substrates are localized to chromatin, some of which are only SUMOylated upon their association with chromatin^9,36–38,42,44–47^. The latter is exemplified by Sgs1 SUMOylation, which is enhanced by Sgs1 association with DNA repair intermediates^37,42^. These observations let us ask whether Smc5/6 may exhibit a better SUMOylation activity in the presence of DNA. We examined double-stranded DNA (dsDNA), single-stranded DNA (ssDNA), and supercoiled circular DNA (SC DNA) (Fig. 2a). We also included DNA repair intermediate structures relevant to Smc5/6 *in vivo* functions, including Holliday Junction (HJ) and dsDNA with a ssDNA tail (ds-ssDNA) (Fig. 2a).

We added each type of DNA at the same nucleotide concentration in SUMOylation reactions containing core-Smc5/6. To better quantify of reactions containing DNA, we used a lower SUMO E3 concentration (25 nM) than those described above (40 nM) without DNA. While all five types of DNA boosted Smc5-Smc6 SUMOylation, HJ and dsDNA showed the greatest effects, with up to 10.5-fold increase of Smc5-Smc6 SUMOylation compared with no DNA control (Fig. 2b–2c) at five minutes. In contrast, ssDNA showed the least potency in stimulation, with no effect seen at five minutes and a 2.8-fold stimulation at ten minutes (Figs. 2b–2c). The addition of ds-ssDNA and supercoiled DNA yielded intermediate effects (Figs. 2b–2c). These data suggest that while multiple types of DNA enhance Smc5/6-mediated SUMOylation, dsDNA and dsDNA-containing structures are superior to ssDNA in stimulation.

### DNA preferentially stimulates Smc5/6 SUMOylation activity relative to Nse2 Smc5

Though Smc5/6 exhibited lower SUMOylation activity compared with Nse2-Smc5 in the absence of DNA, the strong stimulation of its self-SUMOylation in the presence of DNA led us to ask whether Nse2-Smc5 could respond to DNA in a similar manner. Given that HJ conferred a strong simulation of Smc5/6 self-SUMOylation and is a physiological DNA structure for the complex, we focused on this dsDNA-containing structure. We observed that, compared with Nse2-Smc5, the SUMOylation activity of core-Smc5/6 was enhanced to a greater degree by HJ DNA (Fig. 2d). For example, DNA addition could lead to a 12-fold stimulation of the SUMOylation of Smc5-Smc6 when core-Smc5/6 was used as the E3, while less than 2-fold stimulation by DNA was seen when Nse2-Smc5 was the E3 (Fig. 2d, five-minute).

We also examined Sgs1 SUMOylation in the presence of HJ DNA and found that with core-Smc5/6, HJ led to up to a 3.8-fold increase of Sgs1 SUMOylation compared with the no-DNA control (Fig. 2e). A similar effect was also seen for ds-ssDNA (Fig. 2e). We verified that both DNA forms were intact at the timepoint of examination (Supplementary Fig. 2a; Method). While purified Sgs1 unwound ds-ssDNA in helicase reactions containing a critical co-factor, RPA (Supplementary Fig. 2b)^48,49^, the lack of RPA in SUMOylation reactions explains the lack of DNA unwinding therein. We next compared core-Smc5/6 and Nse2-Smc5 side-by-side in SUMOylation reactions. We found that HJ DNA addition resulted in a 3.0-fold increase of Sgs1 SUMOylation when core-Smc5/6 was used, whereas only 1.1-fold stimulation was seen when Nse2-Smc5 was included (Fig. 2f, two-minute). The observation that core-Smc5/6 was better stimulated by DNA than Nse2-Smc5, while the latter exhibited a higher activity without DNA, suggests that as a complex, Smc5/6 can minimize promiscuous SUMOylation in the absence of DNA and achieves better activity in the presence of DNA.

### DNA enables substrate-E3 proximity during SUMOylation

**FIGURE 3.**
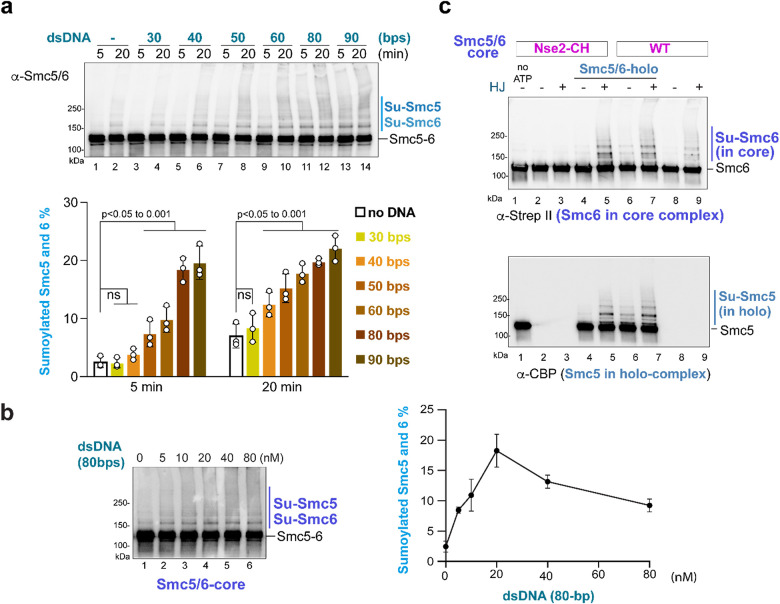
Smc5/6 self-SUMOylation can occur *in trans* in the presence of DNA **a)** DNA length effects on the SUMOylation of Smc5 and Smc6. Experiments were conducted and results are presented as in Figure 2b, except different lengths of dsDNA as indicated were used in the SUMOylation reactions. The percentages of SUMOylated Smc5-Smc6 were quantified and plotted with means, SDs, and p-values indicated (n=3 in all cases). **b)** The effects of DNA concentration on the SUMOylation of Smc5 and Smc6. Experiments were conducted as in panel a, except a lower concentration of core-Smc5/6 (5 nM) was used in the SUMOylation reactions, and a range of dsDNA concentrations were tested. A represented immunoblotting result of 20 min SUMOylation reactions was shown (left) and quantification of three independent experiments is plotted (right) with means and SDs indicated. **c)** Smc5/6 containing an inactive SUMO E3 subunit can be SUMOylated by another Smc5/6 containing active SUMO E3. A representative immunoblot was shown for reactions containing different combinations of Smc5/6 complexes. Details are described in the text.

To explore the mechanisms underlying the stimulatory effect of DNA on Smc5/6-mediated SUMOylation, we asked whether this effect depended on DNA length. Testing dsDNA oligoes ranging from 30- to 90-bp showed that 40-bp DNA was the minimal length required to enhance SUMOylation of Smc5-Smc6, with the strongest effects seen with 80 and 90bp dsDNA (Fig. 3a). A recent cryo-EM structure showed that a single core-Smc5/6 binds to 20-bp dsDNA and Smc5/6 dimers can drive DNA loop formation^21,30^. Thus, a 40-bp minimal DNA length required for stimulation raised the possibility that two core-Smc5/6 complexes may SUMOylate each other *in trans*, with one acting as the E3 and the other as the substrate, when both are bound to the same dsDNA molecule.

The above idea predicts that increasing DNA concentration relative to Smc5/6 may initially enhance SUMOylation by favoring two Smc5/6 complexes localizing to the same DNA molecule. However, this effect can be reversed with a further increase in the DNA to Smc5/6 ratio, as a larger excess of DNA can reduce the chances of two Smc5/6 binding to the same DNA molecule. Upon testing this prediction, the bi-phasic effect was seen. The level of SUMOylated Smc5-Smc6 increased when the DNA: Smc5/6 ratio went from 1:1 to 4:1, followed by a decrease (Fig.3b, right). This observation supports the *trans*-SUMOylation model.

To further test the *trans*-SUMOylation prediction, we generated a mutant core-Smc5/6 wherein the Nse2 E3 residues were mutated (Nse2-CH; C200A, H202A), referred to as core-Smc5/6^Nse2-CH^ (Supplementary Fig. 3)^32,50^. On its own, core-Smc5/6^Nse2-CH^ did not support Smc6 SUMOylation regardless of DNA, as expected (Fig. 3c; lanes 2–3, top). However, when wild-type holo-complex containing CBP-tagged Smc5 was added for 5-min, SUMOylation of Strep II-tagged Smc6 present in the core-Smc5/6^Nse2-CH^ was detected only when DNA was present (Fig. 3c; lanes 4–5, top). A similar observation was made when both complexes were wild-type: SUMOylation of Smc6-Strep II within the core-complex was increased upon the addition of holo-complex (Fig. 3c; lanes 6–9, top). Again, a clear stimulatory Smc6 SUMOylation effect was only seen upon DNA addition. As a control, SUMOylation of Smc5-CBP present in the holo-complex was increased upon DNA addition as expected (Fig. 3c; lanes 4–7, bottom). These data suggest that one Smc5/6 complex can SUMOylate another complex, particularly in the presence of DNA. Collectively, the three results described above provide evidence that DNA can serve the role of bringing two Smc5/6 complexes in proximity of each other to enable *trans*-SUMOylation, though they do not exclude *cis*-SUMOylation wherein Nse2 can SUMOylate subunits within the same complex.

### DNA binding via non-E3 subunits of Smc5/6 enables DNA stimulation of its E3 function

**FIGURE 4.**
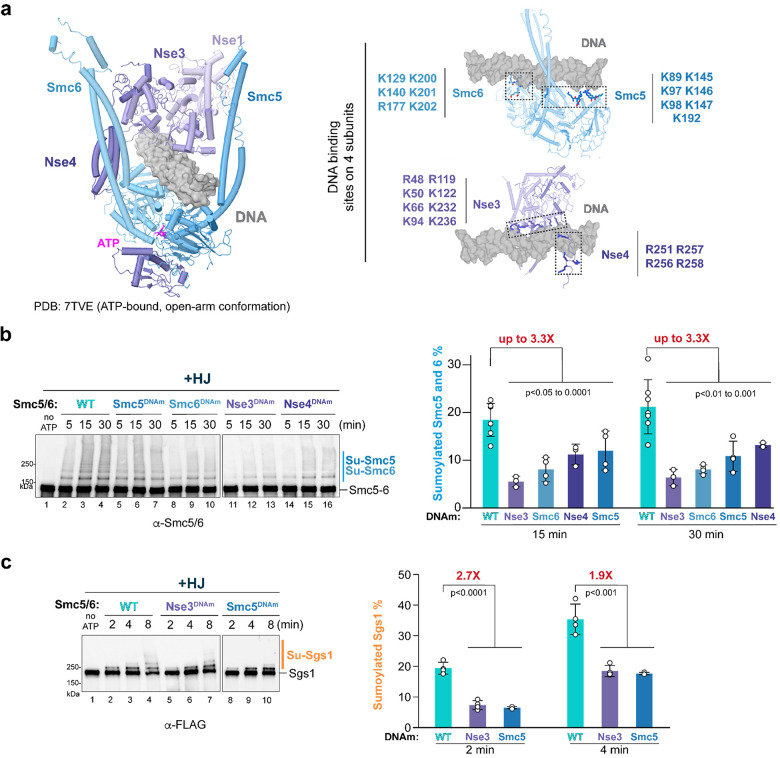
DNA binding sites on non-E3 subunits contribute to DNA stimulation of SUMOylation by Smc5/6. **a)** A cryo-EM structure of Smc5/6 engaged with dsDNA and ATP (PDB: 7TVE) and its DNA binding residues examined in this work^21^. The structure reveals the encirclement of a single dsDNA molecule upon ATP-mediated SMC head dimerization (left). The DNA binding residues are colored according to that of the subunit (right). **b)** Mutating DNA binding residues on Smc5, Smc6, Nse3, or Nse4 reduces the SUMOylation of Smc5-Smc6 in the presence of HJ DNA. (Left) SUMOylation reaction and immunoblotting were examined as in Figure 2b in the presence of HJ DNA, except 100 mM KCl was used in the SUMOylation reaction. Reactions contained wild-type (WT) or DNA binding mutant core-Smc5/6. Right: Percentages of SUMOylated Smc5-Smc6 were derived from at least three independent experiments per condition. Data are plotted as in Figure 2c with means, SDs, and p-values calculated from Welch’s t-test indicated (n=7 for WT; n=4 for Smc5^DNAm^ or Smc6^DNAm^; n=3 for Nse3^DNAm^ or Nse4^DNAm^). Fold-changes indicate different yields of SUMOylated Smc5-Smc6 between wild-type complex and Nse3^DNAm^. **c)** Mutating the DNA binding residues on Nse3 or Smc5 reduces DNA-based stimulation of Sgs1. Experiments were conducted and data are presented as in Supplemental Figure 4c, except 130 mM KCl was used in the SUMOylation reaction. Data are plotted as in Figure 2e with means, SDs, and p-values calculated from Welch’s t-test indicated (n=4 for WT or Nse3^DNAm^; n=3 for Smc5^DNAm^). Fold changes of the percentage of SUMOylated Sgs1 between the WT and the mutant complex are labeled above the bars.

Our data thus far has shown that DNA stimulates SUMOylation reactions when Smc5/6 is used as the SUMO E3. We next investigated which non-SUMO E3 subunits of Smc5/6 with known DNA-binding abilities were required to confer DNA stimulation of SUMOylation. A cryo-EM structure of the core-Smc5/6 revealed a portion of the complex in the dsDNA-bound form (Fig. 4a, left)^21^. In this structure, the SMC head regions are engaged with each other upon ATP binding, while their arm regions are separated, resembling the O-shaped configuration of the complex. A single dsDNA is encircled by the protein ring formed by five subunits, and DNA backbone binding residues were mapped to Smc5, Smc6, Nse3 and Nse4 (Fig. 4a, right)^21^. We thus examined how mutating each of the mapped DNA binding sites affected DNA-based stimulation of SUMOylation. Four mutant core-Smc5/6 complexes, each with altered DNA-contacting residues on one of the DNA-binding subunits, were purified (Supplementary Fig. 4a). The core-Smc5/6 harboring Smc5 DNA binding site mutations (K89, K97, K98, K145, R146, R147, K192 changed to A) is referred to as Smc5^DNAm^ (Fig. 4a). Similarly, the core-Smc5/6 variant harboring DNA binding site mutations on Smc6 (K129, K140, R177, K200, K201, K202 changed to A), Nse3 (R48, K50, K66, K94, R119, K122, K232, K236 changed to A), or Nse4 (R251, R256, R257, R258 changed to A), is referred to as Smc6^DNAm^, Nse3^DNAm^ or Nse4^DNAm^ (Fig. 4a).

The four core-Scm5/6 variants were verified for reduced binding to HJ DNA *in vitro* using standard electrophoretic mobility shift assays (EMSA) (Supplementary Fig. 4b; Left). Apparent dissociation constants calculated for the mutant complexes showed a similar trend in reduction of DNA-binding affinity (Supplementary Fig. 4b; Right). These results, as well as the structural data, suggest that when DNA binding sites are mutated in one of the four subunits involved in DNA binding, the DNA binding sites located in the three other subunits can offer DNA interaction albeit at a reduced level. When tested in SUMOylation reactions, each mutant complex reduced the levels of SUMOylated Smc5-Smc6 and Sgs1 compared with the wild-type core-Smc5/6 and the differences were statically significant (Fig. 4b; Supplementary Fig. 4c). For example, Nse3^DNAm^ and Smc6^DNAm^ led to up to 3-fold reduction of Smc5-Smc6 SUMOylation. We increased the salt concentration to 130 mM (Fig. 4c) from 100 mM (Supplementary Fig. 4c) in reactions to better evaluate mutants effects on Sgs1 SUMOylation, as higher salt concentrations allow better manifestation of DNA binding defects for mutated complexes. We found that Nse3^DNAm^ and Smc5^DNAm^ complexes led to up to a 2.7-fold reduction of Sgs1 SUMOylation in this reaction condition (Fig. 4c). Collectively, these results provide *in vitro* evidence that DNA binding via four non-SUMO E3 subunits contributes to DNA-enhanced Smc5/6 SUMO E3 activity.

### Effects of ATP binding and hydrolysis by Smc5 and Smc6 on the complex’s E3 activity

**FIGURE 5.**
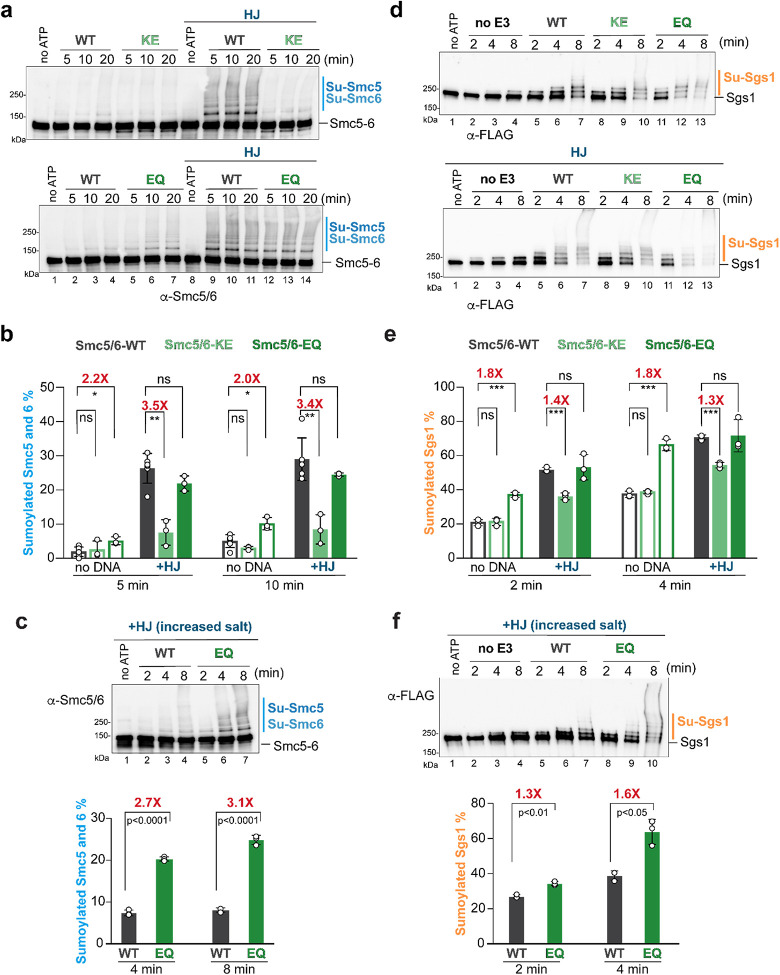
Effects of ATP binding and hydrolysis by Smc5/6 on *in vitro* SUMOylation. **a)** Effects of Smc5/6^KE^ and Smc5/6^EQ^ on the SUMOylation of Smc5-Smc6 in the absence or presence of DNA. SUMOylation reactions contained wild-type or indicated mutant core-Smc5/6 complex as indicated. Experiments were carried out as in Figure 2b. **b)** Percentages of SUMOylated Smc5-Smc6 in SUMOylation reactions. Quantifications of at least three independent experiments per condition are shown as in Figure 2c with means, SDs, and p-values calculated from Welch’s t-test indicated (*p< 0.05; **p < 0.01; ns, not significant; n=6 for WT and WT with HJ; n=3 for other reactions). Fold changes in the percentage of SUMOylated Smc5-Smc6 between WT and mutant complexes are labeled above the bars. **c)** Smc5/6^EQ^ increases the SUMOylation of Smc5-Smc6 in the presence of DNA. Experiments were conducted as in panel a, except a higher salt concentration (110 mM KCl) was used in the SUMOylation reactions. Data are presented as in panel a and b with means, SDs, and p-values calculated from unpaired t-test indicated (n=3). Fold changes in the percentage of SUMOylated Smc5-Smc6 between the WT and mutant complexes are labeled above the bars. **d)** The effects of Smc5/6^KE^ and Smc5/6^EQ^ on Sgs1 SUMOylation in the absence or presence of DNA. Experiments were conducted as panel c, except the inclusion of Sgs1 and Top3-Rmi1 in the SUMOylation reactions. Top: reactions without DNA; bottom, reactions containing HJ. **e)** Percentages of SUMOylated Sgs1 in SUMOylation assays. Quantifications of three independent experiments per condition are shown as in panel b with means, SDs, and p-values indicated (***p < 0.001; ns, not significant; n=3). **f)** Smc5/6^EQ^ increases Sgs1 SUMOylation in the presence of DNA. Experiments were conducted as panel d, except 130 mM KCl was included in the SUMOylation reactions. Data are presented as panel c with means, SDs, and p-values indicated (n=3).

We moved on to examine how ATP binding and hydrolysis by Smc5/6 could contribute to the complex s SUMO E3 activity, either independently or dependently on DNA-mediated effects. To this end, we purified core-Smc5/6 variants, containing mutations at either the ATP binding sites (Smc5/6^KE^; Smc5 ^K75E^ and Smc6^K115E^) or ATP hydrolysis sites (Smc5/6^EQ^; Smc5^E1015Q^ and Smc6^E1048Q^) (Supplementary Fig. 5a)^18,25,30^. As expected, the two mutant Smc5/6 complexes showed impaired ATPase activity regardless of DNA status, while the wild-type Smc5/6 activity was stimulated by DNA (Supplementary Fig. 5b)^18,25^. Compared with the wild-type control, Smc5/6^KE^ reduced Smc5-Smc6 SUMOylation up to 3.5-fold only in the presence of DNA (Figs. 5a top; 5b). No effect by Smc5/6^KE^ was seen in the absence of DNA even when reactions contained a lower salt concentration to allow for more robust SUMOylation (Supplementary Fig. 5c). As Smc5/6^KE^ impairs DNA binding^18,25^, the simplest interpretation of these observations is that ATP-binding by Smc5/6 permits optimal DNA association, thus rendering a stimulatory effect on SUMOylation.

In contrast to Smc5/6^KE^, Smc5/6^EQ^ increased Smc5-Smc6 SUMOylation in the absence of DNA up to 2.2-fold compared with wild-type Smc5/6 (Figs. 5a bottom; 5b; Supplementary Fig. 5c). In the initial tests containing DNA, Smc5-Smc6 SUMOylation produced by the wild-type Smc5/6 were robust and could obscure any potentially positive effects of Smc5/6^EQ^. To address this issue, we examined reactions containing a higher salt concentration to reduce SUMOylation levels (Fig. 5c). We observed that Smc5/6^EQ^ could confer a 3.1-fold increase of Smc5-Smc6 SUMOylation compared with the wild-type control (Fig. 5c). Since Smc5/6^EQ^ stabilizes the complex in the ATP-bound O-shaped conformation regardless of DNA^24^, our data suggest that this conformation could favor Smc5-Smc6 SUMOylation.

The differential behaviors of Smc5/6^KE^ and Smc5/6^EQ^ were also observed for Sgs1 SUMOylation (Figs. 5d–5f). Compared with the wild-type complex, Smc5/6^KE^ only reduced Sgs1 SUMOylation in the presence of DNA and caused no change in the absence of DNA (Figs. 5d–5e). In contrast, Smc5/6^EQ^ boosted Sgs1 SUMOylation up to 1.8-fold in the absence of DNA (Figs. 5d, top; 5e) and up to 1.6-fold in the presence of DNA when higher salt concentration was used to increase SUMOylation stringency (Fig. 5f). Given that each of the two mutant complexes showed the same effect on the SUMOylation of Smc5-Smc6 and Sgs1, we concluded that each altered the complex’s SUMO E3 activity.

### Non-E3 subunits of Smc5/6 are required for Smc5, Smc6 and Sgs1 SUMOylation in cells

Biochemical results described above suggest that non-SUMO E3 subunits of Smc5/6 can directly promote the complex’s SUMO E3 activity by supporting the complex association with DNA and adopting a SUMOylation favorable conformation. We moved on to examine these biochemical conclusions using cellular assays. We first verified that mono-SUMOylated forms of Smc5 and Smc6 could be detected on immunoblots as bands migrating slower than their unmodified forms (Supplementary Figs. 6a–6b)^38,39,51^. While unmodified Smc5 and Smc6 were seen after short exposures of the immunoblots, their SUMOylated forms were visible after longer exposures of the same blots and retarded further when His6-Flag (HF)-tagged SUMO replaced endogenous SUMO (Supplementary Figs. 6a–6b). As shown before, MMS (methyl methanesulfonate) treatment increased poly- or multi-SUMOylated forms of Smc5 and Smc6 (Supplementary Figs. 6a–6b)^39,51,52^.

We found that SUMOylated, but not unmodified, Smc5 forms greatly decreased upon acute depletion of AID degron tagged Smc6, Nse4 of the Nse1-3-4 subcomplex, or Nse6 of the Nse5-6 subcomplex, after the addition of the IAA degron inducer (Supplementary Fig. 6c). This finding is consistent with a previous report showing that mutating several non-SUMO E3 subunits of Smc5/6 reduces Smc5 SUMOylation^35^. Our tests further revealed the same trend for Smc6 SUMOylation in cells depleted of Smc5, Nse4, or Nse6 (Supplementary Fig. 6d). As SUMOylated Sgs1 (su-Sgs1) is induced during recombinational repair upon MMS treatment, we detected its SUMOylation by pulling down SUMOylated proteins using His8-SUMO and probing the immunoblots using an antibody against the Myc-tag fused to Sgs1 (Supplementary Fig. 6e–6f)^37,42^. A reduction in Sgs1 SUMOylation was seen upon depleting Smc5, Nse3, or Nse5 (Supplementary Fig. 6g). The partial reduction seen here matches the effect of the *nse2* SUMO E3 mutant as shown before, reflecting that Sgs1 also undergoes Nse2-independent SUMOylation^36,37^.

### DNA binding sites on Smc5 and Nse4 are critical for Smc5/6-based SUMOylation in cells

Next, we queried whether Smc5/6-mediated SUMOylation could be dampened when its DNA binding sites on non-SUMO E3 subunits were mutated. In a previous study, we constructed alleles of Smc5, Smc6, Nse3, and Nse4 with their DNA binding sites mutated, as done for the DNA binding mutant complexes described above, and showed that only *smc5*^*DNAm*^ and *nse4*^*DNAm*^ cells were viable^21^. Here we examined these two viable mutants for their effects on the *in vivo* SUMOylation of Smc5, Smc6, and Sgs1.

**FIGURE 6.**
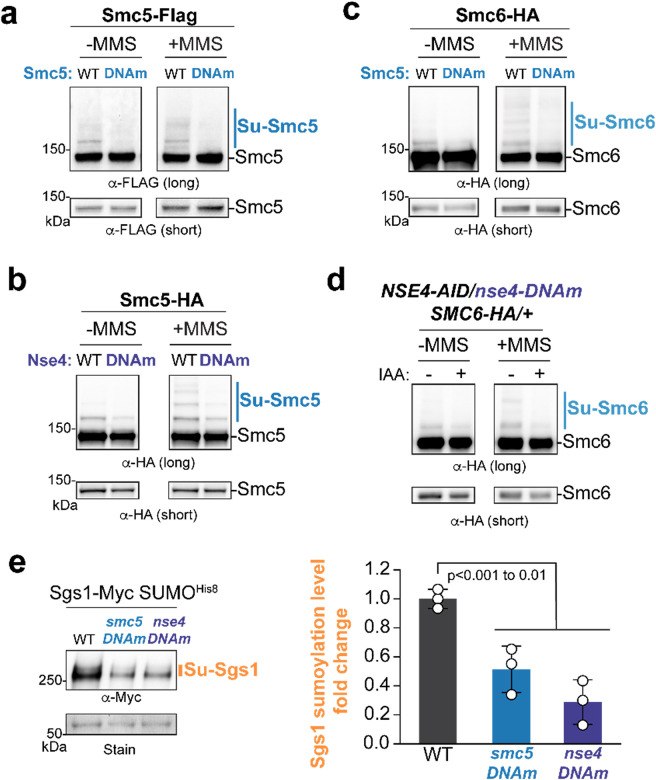
DNA binding mutants impair Smc5, Smc6, and Sgs1 SUMOylation in cells. **a-b)** The effects of DNA binding mutant of *smc5* or *nse4* on Smc5 SUMOylation. Cells containing Smc5 DNA binding residue mutations (*smc5*^*DNAm*^, panel **a**) or Nse4 DNA binding residue mutations (*nse4*^*DNAm*^, panel **b**) with or without MMS treatment (0.03%, 2 h) were examined. Endogenous Smc5 tagged with FLAG (**a**) or 6HA (**b**) in cell extracts was examined using immunoblotting probed with an anti-FLAG or anti-HA antibody. **c-d)** The effects of *smc5* or *nse4* DNA binding mutants on Smc6 SUMOylation. Smc6 SUMOylation was examined with or without MMS treatment as in panel a-b for strains containing *smc5*^*DNAm*^ (**c**) or *nse4*^*DNAm*^ (**d**). Due to lethality caused by combining *nse4*^*DNAm*^ with HA-tagged Smc6, we examined diploid cells containing a copy of *nse4*^*DNAm*^ and another copy of wild-type Nse4 fused to AID that is degraded upon the addition of IAA. **e)** The effects of DNA binding mutants of *smc5* or *nse4* on Sgs1 SUMOylation. Strains contained His8-tagged SUMO and Myc-tagged Sgs1 so that SUMOylated proteins could be enriched using Ni-NTA resins. SUMOylated forms of Sgs1-Myc (su-Sgs1) were detected by immunoblotting using an anti-Myc antibody. Loading is shown by Memcode staining (Stain). Sgs1 SUMOylation was examined in strains with genotypes indicated (left). The percentages of SUMOylated Sgs1 were calculated based on quantification of three independent reactions per condition and are plotted with means, SDs, and p-values indicated (n=3) (right).

Both *smc5*^*DNAm*^ and *nse4*^*DNAm*^ mutants caused a reduction of Smc5 SUMOylation regardless of MMS treatment (Figs. 6a–6b). Similar defects were seen for Smc6 SUMOylation in these mutants (Figs. 6c–6d). As *nse4*^*DNAm*^ caused lethality when combined with HA-tagged Smc6, testing how *nse4*^*DNAm*^ affects Smc6 SUMOylation was done in diploid cells that also contained a wild-type allele of Nse4 fused with the AID degron to induce its acute loss (Fig. 6d). We also examined Sgs1 SUMOylation after MMS treatment in *smc5*^*DNAm*^ and *nse4*^*DNAm*^ cells and found a reduction in both mutants compared with wild-type cells (Fig.6e). These results agreed with *in vitro* data. The stronger SUMOylation defect seen for *smc5*^*DNAm*^ and *nse4*^*DNAm*^ mutants in cells than in biochemical reactions using the mutated complexes can be due to that excess proteins used in the latter could dampen mutants effects. Regardless, the two lines of investigation provide cohesive data to support the conclusion that Nse2-mediated SUMOylation can be supported by non-SUMO E3 subunits of Smc5/6 through DNA binding in cells.

### ATP binding and hydrolysis by Smc5/6 affect its SUMO E3 functions in cells

We next addressed how ATP binding and hydrolysis by Smc5 and Smc6 affect cellular SUMOylation. We generated Smc5^KE^ and Smc5^EQ^ alleles that impair ATP binding and hydrolysis sites of Smc5, respectively. We showed that these HA-tagged alleles caused lethality as expected, in contrast to the HA-tagged wild-type Smc5 that supported growth (Supplementary Figs. 7a–7c)^25,35^. We thus used diploid yeast cells heterozygous for the mutant alleles, so that cell viability could be supported by a wild-type copy of Smc5 fused with the AID degron tag, which permitted IAA-induced Smc5 degradation.

**FIGURE 7.**
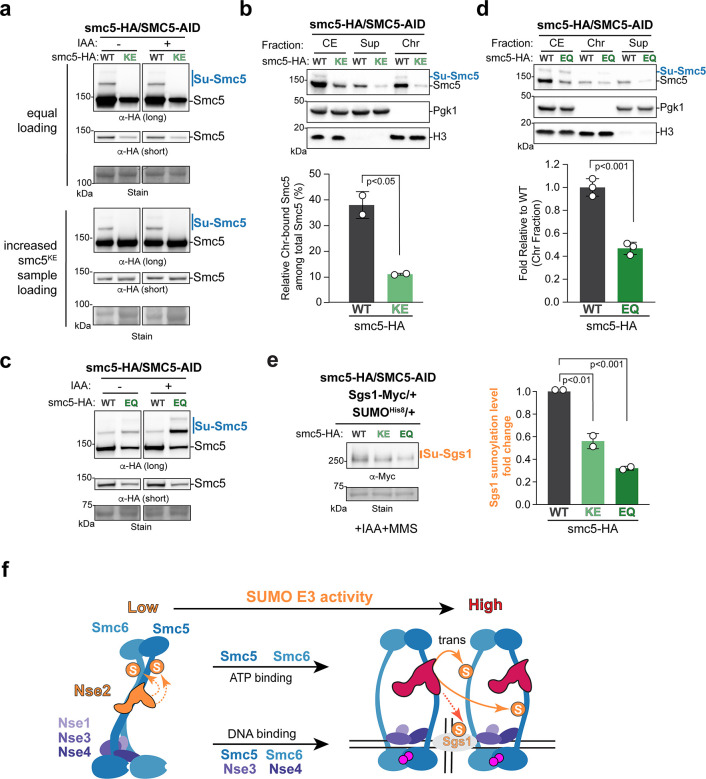
Mutating Smc5 ATP binding and hydrolysis sites affects *in vivo* SUMOylation **a)** Mutating Smc5 ATP binding site abolishes its SUMOylation in cells. SUMOylation of HA-tagged Smc5 was examined and results are presented as in Figure 6b. Acute depletion of the Smc5-AID-Flag protein was achieved by IAA treatment for 90 min. Equal loading is shown in the upper panels and increased loading for Smc5^KE^ samples is shown in the lower panels. **b)** Mutating Smc5 ATP binding site reduces its chromatin association. Smc5 levels in cell extract (CE), chromatin-bound fraction (Chr), and non-chromatin supernatant fraction (Sup) were examined. Top: a representative immunoblotting result showing the detection of HA-Smc5, the chromatin marker H3, and the non-chromatin marker Pgk1. (Bottom) percentages of Smc5 in the chromatin-bound fraction compared with the cell extract after adjusting to H3 levels. Two biological isolates were used in quantification with mean, SD and p-value shown. Unpaired student t-test was used for assessing statistical significance. **c)** Mutating Smc5 ATP hydrolysis site increases its SUMOylation in cells. Smc5 protein SUMOylation was examined and results are presented as in panel a. **d)** The effects of Smc5 ATP hydrolysis site mutant on its chromatin association. Experiments were conducted and data are presented and analyzed as in panel b, except that three biological isolates were used. **e)** Mutating Smc5 ATP binding or hydrolysis site affects Sgs1 SUMOylation in cells. Experiments were conducted and data are presented and analyzed as in Figure 6e, except diploid cells with indicated genotypes were used. **f)** A working model for how Smc5/6 functions as a multi-subunit composite SUMO E3. ATP- and DNA-free Smc5/6 adopts I-shaped conformation. DNA bindings via four subunits and ATP binding via the Smc5 and Smc6 promote the complex association with DNA. The ATP-bound state also drives arm separation, resulting in O-shaped conformation. While DNA can enhance E3 functions by helping to position Smc5/6 near its SUMOylation substrates (another Smc5/6 or Sgs1), ATP binding induced O-shaped conformation favors SUMO transfer to substrates.

Strikingly, SUMOylation was undetectable for HA-Smc5^KE^ but readily seen for the HA-Smc5 control, regardless of Smc5-AID degradation (Fig. 7a, top). This effect was also seen upon adjusting loading to match wild-type and mutant Smc5 protein levels (Fig. 7a, bottom). Agreeing with *in vitro* findings that Smc5/6^KE^ is defective in DNA association^25^, the percentage of Smc5^KE^ associated with chromatin was reduced compared with that of the wild-type protein (Fig. 7b). Thus, both *in vivo* and *in vitro* data suggest that ATP-binding promotes Smc5 SUMOylation at least partly via enabling Smc5/6 engagement with DNA and chromatin.

In contrast to Smc5^KE^, the SUMOylation level of HA-Smc5^EQ^ was higher than the HA-Smc5 control, regardless of Smc5-AID degradation (Fig. 7c). This is consistent with biochemical data that Smc5/6^EQ^ enhances Smc5-Smc6 SUMOylation (Fig. 5a–5c; Supplementary Fig. 5c). The combined *in vitro* and *in vivo* data suggest that the ATP-hydrolysis mutant of Smc5/6, which favors O-shaped conformation, can enhance SUMOylation. In the meantime, we found that Smc5^EQ^ reduced chromatin association compared with the wild-type Smc5 (Fig. 7d). This result predicts that while Smc5^EQ^ may favor a complex conformation more potent for SUMO E3 activity, its reduced chromatin association could hinder the ability to encounter substrates that are not Smc5/6 subunits. Indeed, Smc5^EQ^ reduced Sgs1 SUMOylation in cells as did Smc5^KE^ (Fig. 7e). These results suggest that ATP-binding and hydrolysis by Smc5/6 influence cellular SUMOylation via affecting both its chromatin association and conformations (see Discussion).

## DISCUSSION

SUMO E3 enzymes play important roles in enabling SUMOylation efficiency and specificity. In this work, we examined how the multi-subunit Smc5/6 complex containing the Nse2 E3 subunit promotes SUMOylation. We found that core- and holo-Smc5/6 exhibited E3 activity *in vitro*, and this was stimulated by multiple types of DNA, with dsDNA conferring stronger stimulation than ssDNA. Our data further suggested that the observed DNA stimulative effect could stem from enhancing substrate-enzyme proximity. Moreover, this effect required DNA binding activities of four non-SUMO E3 subunits as well as ATP binding by the two SMC subunits. Finally, we found that while the O-shaped conformation adopted by the ATP-bound form of Smc5/6^EQ^ favored its E3 activity, it reduced chromatin association of Smc5/6, thus dampening the SUMOylation of non-Smc5/6 proteins. Taken together, our findings provide a mechanistic framework for how a multi-subunit SUMO E3 complex harnesses the different activities of its subunits to achieve efficient SUMOylation of chromatin-bound substrates.

### Mechanisms of dsDNA-based stimulation of Smc5/6 SUMOylation activity

Nse2 E3 s substrates are chromatin-associated proteins, some of which have been shown to be SUMOylated only upon association with DNA^9,36–38,42,44–47^. To address how Nse2 specifically targets this pool of substrates, we examined core- or holo-Smc5/6 in SUMOylation reactions. We show that both forms of Smc5/6 complexes act as SUMO E3s *in vitro* (Figs. 1c–1d). Significantly, while they were less potent than the Nse2 Smc5 dimer without DNA, their activity increased up to 10-fold in the presence of DNA, far exceeding the enhancement seen for Nse2 Smc5 (Figs. 2b–2f). These data suggest that the Smc5/6 complex can achieve efficient SUMOylation in the presence of DNA while reducing promiscuous modification without DNA. We note that while ssDNA was reported to be superior to dsDNA in enhancing Nse2-Smc5’s activity^34^, the converse is found for Smc5/6 as shown in Figure 2. Since Nse2 is an obligatory subunit of Smc5/6 and co-purifies with all seven other subunits^8–13^, it is possible that Nse2-Smc5 in isolation may gain an ability to better interact with ssDNA. Overall, the observations that dsDNA and dsDNA-containing structures can serve as an effective SUMO E3 stimulative agent for the complete Smc5/6 complex provides one explanation for its preferential SUMOylation of chromatin-associated substrates in cells.

Our mutagenesis data further revealed that DNA binding sites located on four non-E3 subunits contribute to DNA stimulated SUMOylation by the core-Smc5/6 complex *in vitro* (Figs. 4b–4c, Supplementary Fig. 4c). Consistent with this finding, optimal SUMOylation of Smc5, Smc6 and Sgs1 in cells also requires DNA binding sites of Smc5 and Nse4 (Fig. 6). Examining self-SUMOylation of Smc5 and Smc6, we found that a minimal DNA length capable of accommodating two Smc5/6 complexes was required for DNA-based stimulation of SUMOylation (Fig. 3a). Further, the SUMO E3 dead Smc5/6 (core-Smc5/6^Nse2-CH^) was efficiently SUMOylated by the wild-type Smc5/6 only in the presence of DNA, suggesting that DNA enhances SUMOylation *in trans* (Fig. 3c). A bi-phasic effect of DNA concentration was detected, wherein initial increase of the DNA:Smc5/6 ratio boosted SUMOylation but further increase of ratio reduced SUMOylation (Fig. 3b). The simplest explanation of the combined biochemical results is that DNA binding can position two Smc5/6 complexes in proximity, thus allowing one (E3) to SUMOylate the other (substrate). We envision that DNA stimulation of Sgs1 SUMOylation by Smc5/6 is also mediated by bringing the E3 in proximity with the substrate. Whether ssDNA interaction with hinge and arm regions of Smc5-Smc6^20,34,53^ may contribute to ssDNA-mediated regulation of Smc5/6 SUMOylation awaits future exploration.

### Mechanisms of ATP-based promotion of the SUMO E3 function of Smc5/6

Our data suggest that ATP regulates Smc5/6 SUMO E3 functions via a dual effect. It is known that Smc5 and Smc6 binding ATP molecules between their head regions promotes the complex association with DNA as well as induces large conformational changes^18,21–24^. Examination of ATP binding and hydrolysis mutants of Smc5/6 suggest that both effects of ATP appear to influence SUMOylation. First, the ATP-binding mutant Smc5/6^KE^, known to reduce DNA association^25^, impaired SUMOylation only in the presence of DNA *in vitro* (Figs. 5a–5b; 5d–5e). This result suggests that ATP binding by Smc5/6 contributes to its SUMO E3 function partly via promoting complex-DNA association. This conclusion is supported by cellular data that Smc5^KE^ reduced its association with chromatin and the SUMOylation of both itself and Sgs1 (Figs. 7a, 7b, 7e).

Second, studies of the ATP hydrolysis mutant of Smc5/6 (Smc5/6^EQ^) in the absence of DNA revealed another facet of the effect of ATP on SUMOylation, that is, Smc5/6 stabilized in ATP-bound conformation intrinsically favors its E3 activity (Figs. 5a–5f; Supplementary Fig. 5c). While Smc5/6^EQ^ is mainly in an O-shaped conformation, the ATP-free complex is predominantly in an I-shaped conformation^18–24^. In the I-shaped conformation, Nse2 binds to both Smc5 and Smc6 arm regions that zip up together, whereas in the O-shaped conformation, Nse2 is freed from the Smc6 arm as the two arm regions dissociate^18–24^. In comparison with the I-shaped conformation, the O-shaped conformation, in principle, can provide more structural flexibility and space for Nse2 to interact with the SUMO E2 and substrates. Both these factors can favor the complex’s E3 functions.

In addition, our analyses of published structures unveiled that I- and O-shaped conformations of Smc5/6 can differentially affect the C-terminal SUMO interaction motif (SIM) of Nse2 (Supplementary Fig. 8). When Nse2 only binds to Smc5 as seen in the O-shaped configuration, its C-terminal SIM can engage with the SUMO molecule at the backside of the Ubc9 E2, thus enhancing E3 activity (Supplementary Fig. 8a)^33^. However, in the I-shaped configuration, this SIM region adopts a helical structure and engages the Smc5 arm region (Supplementary Fig. 8b)^23^. As such, alternation of Nse2 C-terminal SIM may provide another explanation for the more potent SUMO E3 activity of Smc5/6^EQ^, which adopts mainly the O-shaped conformation (Supplementary Figs. 8c–8d). Cellular studies confirmed that Smc5^EQ^ showed enhanced SUMOylation of itself (Fig. 7c). However, diminished chromatin association of Smc5^EQ^ in cells (Fig. 7d) can reduce the complex encountering Sgs1 on DNA for SUMOylation (Fig. 7e). Further testing of the influence of distinct Smc5/6 conformations on SUMOylation awaits single molecular and structural analyses and could provide deeper insight into the Smc5/6 E3 functions.

### A working model for how Smc5/6 achieves SUMOylation specificity and efficiency

Integrating our results with previous findings, we propose a model for the mechanisms underlying the SUMOylation functions of Smc5/6 (Fig. 7f). We suggest that Smc5/6 binding to dsDNA and chromatin, which is partly mediated by its four non-SUMO E3 subunits (Smc5, Smc6. Nse3, and Nse4) and is favored by SMC binding to ATP, enhances Nse2-mediated SUMOylation by bringing the enzyme in proximity with DNA-bound substrates. ATP-binding also renders an O-shaped conformation of Smc5/6 that favors Nse2’s SUMO E3 activity, likely by allowing more efficient engagement with SUMO, E2, and substrates. As such, multi-subunit collaboration can provide a means to increase SUMOylation efficiency and specificity. This model offers explanation for previous findings that SUMOylation of Smc5/6 substrates such as Sgs1 is enhanced by the generation of DNA repair intermediates^37,42^. Our conclusions also support a shared principle with multi-subunit ubiquitin E3 complexes, which also utilize intricate collaboration among all non-E3 subunits to achieve modification efficiency and specificity^54–56^.

The loss of substrate SUMOylation upon acute depletion of Smc5/6 subunits in cells (Supplementary Figs. 6c, 6d, 6g) can reflect simultaneous alteration of the complex’s conformation as well as its DNA and ATP binding cycles. While the full picture of how each subunit integrates its multiple biochemical activities in regulating SUMOylation is to be completed, our work provides a critical step toward this goal by unveiling how DNA and ATP binding by several subunits can render SUMOylation specificity and efficiency. We note that Smc5/6 can also use additional mechanisms that are not addressed in this work to support SUMOylation in cells. For example, previous studies suggested that SUMO interaction motifs located on Nse5 can help Smc5 and Smc6 SUMOylation in cells, likely by enriching local SUMO concentration^19,57^. This feature was not explored *in vitro* as abundant SUMO in the SUMOylation reactions can mask this effect. Additional attributes of Smc5/6, such as its interactions with substrates and localization at specific regions of the genome, can also influence SUMOylation efficiency and specificity in cells. As the SUMO E3 function of Smc5/6 facilitates almost all processes that this complex contributes to, such as DNA replication, DNA repair and viral restriction^12,13^, this work lays a foundation for future research to gain more insights into how Smc5/6 enables SUMOylation at specific times and locations upon unique substrates to maximize genome maintenance.

## METHODS

### Yeast strains and genetic methods.

Strains used in this study are listed in Supplementary Table 1 and are isogenic to W1588–4C, a *RAD5* derivative of W303 (*MATa ade2-1 can1-100 ura3-1 his3-11,15, leu2-3, 112 trp1-1 rad5-535*)^9^. At least two strains per genotype were examined for each assay, and one is listed in Supplementary Table 1. Protein tagging and mutant construction were conducted using standard PCR-based methods. All genetically altered loci were verified by sequencing. Standard procedures were used for cell growth, media preparation, and tetrad analyses. To degrade AID-tagged protein, 1 mM IAA (Indole-3-acetic acid, Sigma) was added to asynchronous cultures for 90 min as previously described^58^. Cells were grown at 30 °C in YPD media in all experiments, and MMS treatments were conducted with 0.03% MMS (Sigma) for 2 hours.

### Plasmids expressing mutant forms of Smc5/6.

All plasmids used in this work are included in Supplementary Table 2. The pET-expression plasmid contains the *Saccharomyces cerevisiae* genes encoding Smc5, Smc6, and the Nse1-4 proteins, with Smc6 fused with the 3C-Twin-Strep tag^18^. This plasmid was used to generate constructs containing each of the four DNA binding site mutants, and the ATP binding or hydrolysis mutants; mutated residues are included in the Results section. In each case, the synthesized DNA fragment containing the indicated mutations was exchanged with the corresponding wild-type gene using the NEB Gibson-Assembly Cloning Kit (NEB E5510S). All plasmids were confirmed by sequencing.

### Purification of core-Smc5/6 complex.

Expression and purification of the wild-type core-Smc5/6 were carried out following a published protocol^21^ with a few modifications. The same method was also used for obtaining mutant forms of the complex. Briefly, a pET-Smc5/6-hexamer plasmid expressing either the wild-type or a mutant form of the core complex was transformed into *E. coli* BL21 (DE3) cells. Cells were grown in 2 L of TB-medium at 37 °C to reach *A*_600_ 1.0. Protein expression was induced by 0.4 mM IPTG at 22°C for 16 h. Cell pellets were collected, and all subsequent steps were carried out at 4°C. First, cell pellets were resuspended in 50 mL of lysis buffer (50 mM Tris-HCl pH 7.5, 300 mM NaCl, 5% glycerol, 25 mM imidazole) supplemented with 5 mM DTT, 1 mM PMSF and 150 U Benzonase (EMD Millipore), followed by sonication at 40 Amplitude for 15 min with pulsing using 1 s on and off cycle. Cell lysate was clarified by centrifugation (100,000 g, 45 min) before passing through a 0.45 μm filter. Cleared lysate was applied to a 5 mL Strep-Tactin (IBA Lifesciences) column, pre-equilibrated with 25 mL of lysis buffer supplemented with 2 mM DTT. The column was washed with 50 mL the same buffer, and proteins were eluted with 20 mL of lysis buffer supplemented with 2 mM DTT and 2.5 mM desthiobiotin. Eluates were applied to a 5 mL HiTrap Heparin column (Cytiva), pre-equilibrated with 10 mL lysis buffer containing 2 mM DTT. The column was washed with 25 mL of the same buffer before eluted with 20 mL of Heparin elution buffer (20 mM Tris pH 7.4, 1 M KCl, 10% glycerol, 0.5 mM EDTA, 0.01% Igepal, 1 mM DTT). Peak fractions containing core-Smc5/6 were concentrated using Amicon Ultra centrifugal filter units (50 kDa cutoff) before loading to a 24 mL Superose 6 10/300 GL size-exclusion chromatography column (Cytiva) in storage buffer (20 mM Tris pH 7.4, 250 mM KCl, 10% glycerol, 0.5 mM EDTA, 0.01% Igepal, 1 mM DTT). The peak fractions containing core-Smc5/6 were concentrated to 1–2 μM, and stored in aliquots at −80°C.

### Purification of holo-Smc5/6 complex.

A previously described protocol was used, and it is briefly described below^19^. Each subunit was expressed under the inducible galactose promoter in yeast cells, with Smc5 fused with a CBP tag. Cells grown at 30 °C in YP media containing 2% glycerol and 2% lactic acid were supplemented with 2% galactose to induce protein expression. Cell pellets were resuspended in buffer E (45 mM HEPES-KOH pH 7.6, 10% glycerol, 0.02% NP40) supplemented with 100 mM NaCl, 1 mM DTT, Protease inhibitor cocktail (Sigma), and cOmplete^™^ Ultra EDTA free protease inhibitor (Roche). Cell pastes were frozen dropwise in liquid nitrogen and broken in a freezer mill (SPEX CertiPrep 6850 Freezer/Mill). The resultant powders were resuspended with buffer E supplemented with 300 mM NaCl and 1 mM DTT before centrifugation to remove debris. The lysate was supplemented with 2 mM CaCl_2_ and incubated with calmodulin resin for 2 h at 4°C. Resins were washed with 10 bed volume of buffer E supplemented with 300 mM NaCl, 2 mM CaCl_2_ and 1 mM DTT, and proteins were eluted using the same buffer without CaCl_2_ but containing 1 mM EDTA and 2 mM EGTA. Peak fractions were pooled and subjected to gel filtration on a Superose 6 Increase column. Fractions containing holo-Smc5/6 were collected and snapped frozen for storage at −80°C.

### Purification of SUMOylation enzymes, Sgs1, Top3-Rmi1, and the Nse2-Smc5 dimer.

Expression and purification of the budding yeast SUMO, the SUMO E1 (Aos1-Uba2), the SUMO E2 (Ubc9), The Nse2-Smc5 complex, FLAG-Sgs1, the V5-Top3 and GST-Rmi1 complex were carried out following published procedures^32,42,48,59^.

### DNA preparation and EMSA assay.

Standard procedures were used to anneal oligonucleotides to form HJ (40-mer each arm), dsDNA, and ss-dsDNA (40-mer duplex with a 3’ 40-mer ssDNA overhang). The annealed products were verified by gel electrophoresis. Sequences of the oligonucleotides used in this work are listed in Supplementary Table 3. pBlueScript (+) plasmid extracted from *E. coli* cells was used as supercoiled (SC) DNA in SUMOylation reactions. For EMSA assay, H3 oligo was first labeled with an IRDye 680RD (LICORbio) at its 3 end before annealing with oligo H5, H7 and H8. The resulting fluorescent labeled HJ substrate was purified by gel electrophoresis. Wild type core-Smc5/6 or its DNA binding mutants (80–240 nM) were incubated with the fluorescent labeled HJ substrate (60 nM) in 10 μL of buffer D (20 mM Hepes-Na, pH 7.5, 150 mM NaCl, 1 mM ATP, 1 mM DTT) for 30 min at 4°C, followed by incubation at 30°C for 30 min. The reaction mixtures were resolved in 0.8% agarose (ThermoFisher, catalog: BP2410) gels in 0.5xTBE buffer (22.5 mM Tris-borate at pH 8.0, 0.5 mM EDTA). Gel electrophoresis was carried out at 50 V for 3h at 4°C. Gels were dried and analyzed using an Amersham Typhoon 5 Biomolecular Imager. The percentages of HJ DNA shifted by proteins were quantified using the ImageQuant TL software and fitted to a single linear regression curve using GraphPad Prism. The apparent K_d_ was calculated as the protein concentration with 50% of HJ binding observed.

### *In vitro* SUMOylation assays.

Standard SUMOylation reactions were carried out as described previously^42^ in buffer R (45 mM HEPES-Na pH 7.0, 5 mM MgCl_2_, 0.1 mM DTT) supplemented with 70 mM KCl in a total volume of 20 μL. Reactions contained the SUMO E1 complex Aos1-Uba2 (50 nM), the SUMO E2 enzyme Ubc9 (280 nM), the yeast SUMO Smt3 (2.2 μM), with or without Sgs1 and Top3-Rmi1 proteins (each at 30 nM). DNA was added at 12.8 μM nucleotides concentration, unless noted otherwise. We note that Smc5/6 interacts with dsDNA and ssDNA at apparent dissociation constants of approximately 100 nM^18^, which is close to the DNA concentration used in the SUMOylation reactions. For single SUMO E3 reactions, E3 was added at 40 nM; for reactions containing two types of SUMO E3s, each was added at 25 nM. Reactions testing DNA as a stimulative agent contained 25 nM SUMO E3, while those testing DNA concentration dependency contained 5 nM Smc5/6. The SUMOylation reactions were placed on ice for 10 min before being initiated by the addition of 5 mM ATP and incubating at 30°C. Samples were taken at indicated time points, mixed with sample loading buffer, and denatured at 95°C for 2 min. Samples were analyzed by SDS-PAGE and immunoblotting. To facilitate the quantification of Sgs1 SUMOylation, its poly- and multi-SUMOylation was reduced by including 100 mM KCl (Figs. 2e, 2f; Supplementary Fig. 4c) or 130 mM KCl (Fig. 4c) in the SUMOylation reactions. Smc5/6^EQ^, together with WT or Smc5/6^KE^, was tested in both standard reaction conditions, and those contained 110 mM KCl (Figs. 5c, 5d), 130 mM KCl (Fig. 5f) or 40 mM KCl (Supplementary Fig. 5c). The reactions comparing core-Smc5/6 and Nse2-Smc5 E3 were carried at both 70 mM (Fig. 2d) and 100 mM KCl (Fig. 2f) reaction conditions. While studying the complete Smc5/6 complex and the Sgs1-Top3-Rmi1 (STR) complex provide insights into physiologically relevant SUMOylation, low concentrations of these protein preparations prevented detailed kinetic analyses.

### Examining DNA status in SUMOylation reactions.

To determine whether HJ or ds-ssDNA were unwound by Sgs1 under the SUMOylation reaction conditions (Fig. 2e), the SUMOylation reactions were stopped and deproteinized by treatment with SDS (0.1%) and proteinase K (0.5 mg/mL) for 10 min at 37°C. The samples were then resolved on 7% polyacrylamide gels in TAE buffer (40 mM Tris, 20 mM Acetate acid and 1 mM EDTA) at 4°C. HJ DNA in the reactions was detected by ethidium bromide (EtBr) staining, The ds-ssDNA structure generated by annealing the oligo H3 with an IRDye 680RD labeled H2 oligo (Table S2) was detected by fluorescent scanning on an Amersham Typhoon 5 Biomolecular Imager. A heat denatured HJ or ds-ssDNA was included to indicate the unwinding products.

### Sgs1-Top3-Rmi1 helicase assay.

Sgs1 and Top3-Rmi1 (20 nM or 40 nM) were incubated with the fluorescent labeled ds-ssDNA substrate (5 nM) in the presence of yeast RPA (40 nM) in 10 μL of buffer (25 mM Tris-HCl, pH 7.5, 1 mM DTT, 100 μg/mL BSA, 2 mM MgCl_2_, 2 mM ATP, and the ATP regenerating system) at 37°C for 30 min. The reaction was stopped by treatment with SDS (0.1%) and proteinase K (0.5 mg/ml) for 5 min at 30°C. The reaction mixtures were resolved on a 7% polyacrylamide gel in TAE buffer at 4°C. Gels were dried onto Hybond-N+ positively charged nylon transfer membrane (Cytiva) and then analyzed in an Amersham Typhoon 5 Biomolecular Imager.

### ATPase assay.

Wild type core-Smc5/6 complex and its mutant forms (100 nM) were incubated with or without an 80-mer dsDNA (1 μM) in 10 μl buffer (30 mM Tris-HCl, pH 7.5, 2.5 mM MgCl_2_, 1 mM DTT, 100 μg/ml BSA, 80 mM KCl) that also contained 0.5 mM ATP and 25 nCi [γ-^32^P]-ATP. Reactions were conducted at 30 °C and samples at indicated time points were analyzed by thin layer chromatography (TLC) followed by phosphorimaging^60^.

### Detection of cellular protein SUMOylation.

To detect SUMOylation of Smc5 and Smc6, protein extracts were made using a TCA (trichloroacetic acid) method as previously described^61^. In brief, cell pellets were resuspended in 20% TCA and homogenized using glass beads in a FastPrep-24 bead beating instrument (MP Biomedicals). The lysate was centrifuged to remove the supernatant. The precipitated proteins were dissolved in Laemmli buffer (65 mM Tris-Cl pH6.8, 2% SDS, 10% glycerol, 5% β-mercaptoethanol, and 0.025% bromophenol blue) with 2 M Tris to neutralize the lysate. Prior to loading, samples were boiled for 5 min and spun down at 13,200 × g for 5 min to remove insoluble materials. Samples were separated on NuPAGE^™^ 3– 8% Tris-acetate gels (Thermo Fisher EA03752) for immunoblotting to detect both SUMOylated and unmodified Smc5 or Smc6.

To detect SUMOylation of Sgs1, a denaturing SUMO pull down method was used wherein protein was extracted in denaturing conditions to minimize deSUMOylation^62^. In brief, cells containing His8-tagged SUMO were mixed with 55% TCA and then buffer A (6 M guanidine HCl, 100 mM sodium phosphate at pH 8.0, 10 mM Tris-HCl at pH 8.0). The solution was incubated with Ni-NTA resin (Qiagen 30210) in the presence of 0.05% Tween-20 and 4.4 mM imidazole with overnight rotation at room temperature. Beads were washed twice with buffer A supplemented with 0.05% Tween 20 and then four times with buffer C (8 M urea, 100 mM sodium phosphate at pH 6.3, 10 mM Tris-HCl at pH 6.3) supplemented with 0.05% Tween 20. Proteins were eluted from the beads using HU buffer (8 M urea, 200 mM Tris-HCl at pH 6.8, 1 mM EDTA, 5% SDS, 0.1% bromophenol blue, 1.5% DTT, 200 mM imidazole). Samples were loaded onto NuPAGE^™^ 3– 8% Tris-acetate gels (Thermo Fisher EA03752) for immunoblotting to detect SUMOylated Sgs1. Equal loading was verified by using Pierce Reversible Protein Stain Kit for Nitrocellulose Membranes (Thermo Fisher 24580).

### Chromatin fraction assays.

Chromatin fractionation was carried out as described previously^21^. Log-phase growing yeast cells were harvested and processed to generate spheroplast using purified lyticase. This was followed by cell lysis in extraction buffer (20 mM Pipes-KOH pH 6.6, 150 mM KOAc, 2 mM Mg(OAc)_2_,1 mM NaF, 0.5 mM Na_3_VO_4_, 11% Triton X-100) supplemented with protease inhibitor mixture (Sigma P8215) for 5 min on ice. Lysates were cleared by centrifugation at 16,000 g for 15 min on a sucrose cushion. Chromatin pellets were recovered after washing with the extraction buffer and resuspended in the same buffer. Protein fractions were mixed with gel loading buffer and boiled for 5 min. Samples were subjected to electrophoresis on 4–20% Tris-glycine gels (Bio-Rad 4561096) and immunoblotting.

### Immunoblotting analysis and antibodies.

To assess *in vitro* and *in vivo* protein SUMOylation, proteins were separated by SDS-PAGE and transferred onto a 0.2 μm nitrocellulose membrane (GE, #G5678144) for immunoblotting. Antibodies used were anti-Flag (M2, F1804, Sigma, 1: 1000 dilution), anti-TAP (PAP, P1291, Sigma, 1: 5000 dilution), anti-Myc (9E10, BE0238, Bio X Cell, 1: 1000 dilution), anti-HA (3F10, 12158167001, Roche, 1:1000 dilution), anti-H3 antibody (ab1791, Abcam 1:1000). anti-Pgk1 antibody (22C5D8, Invitrogen, 1:5000), and a customized rabbit antibody raised against purified holo-Smc5/6 complex, referred to as pan-Smc5/6 antibody or -Smc5/6 (Pocono Rabbit Farm & Lab, PA, 1:1000 dilution). For *in vivo* SUMOylation detection, immunoblots were developed with ECL+ (Bio-Rad) and signals were detected using a Fujifilm LAS-3000 luminescent image analyzer. To analyze *in vitro* SUMOylation, immunoblots were developed with Clarity Western ECL Substrate (Bio-Rad) and signals were visualized using a Chemidoc Imager (Bio-Rad). Both image analyzers have a linear dynamic range of 10^4^. Signal intensities of non-saturated bands were quantified using ImageJ or ImageQuant^™^ TL software.

### Quantification and statistical analysis.

Sample size and presentations are reported in the figure legends. *P* values were determined from two-tailed unpaired Student t-tests when sample sizes are the same. When sample sizes are different, *p* values were determined from Welch’s t-tests and indicated in figure legends (*p<0.05; **p<0.01; ***p< .001; ****p<0.0001).

## Supplementary Material

1

## Data Availability

Data supporting the findings of this study are available within the article, the accompanying source data files, and the Supplementary Information. Source data are provided with this paper.
