# Supplementary material for: Multi-subunit collaboration enables Smc5/6 to function as a composite SUMO E3 complex
Source: Res Sq. 2026 Apr 30:rs.3.rs-9557402. Preprint. [Version 1] doi: 10.21203/rs.3.rs-9557402/v1 (PMC13142630; doi:10.21203/rs.3.rs-9557402/v1)
Supplement: 1 [file NIHPPrs9557402V1-supplement-1.pdf]

Supplementary Fig. 1. Purified proteins used for *in vitro* studies.

a) The budding yeast Sgs1, Top3-Rmi1 complex, SUMO (Smt3), the SUMO E1 (the Aos1-Uba2 complex), and the SUMO E2 (Ubc9) were purified and analyzed by 4-20% gradient SDS-PAGE. A Coomassie blue-stained gel picture is shown, with each protein labeled.

b-c) Smc5 and Smc6 are SUMOylated within the Smc5/6 complex. (b) SUMOylation of the CBP-tagged Smc5 within the holo-Smc5/6 complex was detected after the SUMOylation reactions were examined by immunoblotting probed with anti-CBP antibody. (c) SUMOylation of Strep II-tagged Smc6 within the core-Smc5/6 complex was detected after the SUMOylation reactions were examined by immunoblotting probed with anti-Strep II antibody. HJ DNA was added to both types of SUMOylation reactions.

Supplementary Fig. 2. DNA structures in the SUMOylation reactions and Sgs1 activity assays.

a) SUMOylation reactions were deproteinized and examined using 7% polyacrylamide gels to examine HJ DNA and ds-ssDNA included in the reactions. (Left) HJ DNA in the indicated reactions and denatured HJ structure as a control (lane 2) were examined to show that HJ DNA remained intact in SUMOylation reactions. (Right) ds-ssDNA was fluorescent labeled (red dot) in the SUMOylation reactions to better separate them from the unwound products (lane 7) to show that ds-ssDNA in the SUMOylation reactions were intact.

b) Sgs1 unwinds the fluorescent labeled ds-ssDNA in the presence of yeast RPA under the helicase assay conditions.

Supplementary Fig. 3. Purified wild-type and Nse2-CH mutant core-Smc5/6 complexes.

A Coomassie blue stained gel picture of core-Smc5/6 containing either wild-type Nse2 or Nse2-CH mutant protein. Nse2-CH mutant protein contains its SUMO E3 mutations (C200A, H202A).

Supplementary Fig. 4. Smc5/6 DNA binding mutant characterization.

a) Purified wild-type core-Smc5/6 and core-Smc5/6 complexes mutated for DNA binding sites. Mutant core-Smc5/6 complexes include those affecting the DNA binding sites on Smc5 ($Smc5^{DNAm}$), Smc6 ($Smc6^{DNAm}$), Nse3 ($Nse3^{DNAm}$), and Nse4 ($Nse4^{DNAm}$). Complexes were purified and analyzed by SDS-PAGE on 4-20% gradient NuPAGE Bis-Tris gels followed by staining with Coomassie blue.

b) Four DNA binding mutants of Smc5/6 are compromised for HJ binding. One example of three independent repeats of EMSA assays was shown. The apparent K_d for each complex was calculated as the complex concentration causing 50% of HJ binding, with means, SDs, and p-values indicated (n=3 for each complex).

c) Mutating DNA binding sites on Smc5, Smc6, Nse3, or Nse4 reduces Sgs1 SUMOylation in the presence of DNA. Experiments were conducted as in Figure 2e using wild-type or mutant core-Smc5/6 as indicated (Top). Percentages of SUMOylated Sgs1 derived from at least three independent experiments per condition were plotted as in Figure 2e with means, SDs, and p-values calculated from Welch’s t-test indicated (n=4 for WT; n=3 for each mutant) (Bottom).

Supplementary Fig. 5 Characterization of Smc5/6 variants with ATP-binding or hydrolysis sites mutated.

a) A Coomassie blue stained gel picture of purified ATP binding and hydrolysis mutant core-Smc5/6. Purified wild-type core-Smc5/6 along with mutant complex with either the ATP binding sites (Smc5/6^{KE}; KE) or the ATP hydrolysis sites (Smc5/6^{EQ}; EQ) mutated were examined as Supplemental figure 4a.

b) Wild-type core-Smc5/6, but not Smc5/6 variant with its ATP binding site or ATP hydrolysis site mutated, supports ATPase activity. Quantification shows the means, SDs, and p-values (n=3 independent tests per condition). As shown previously, the ATPase activity of wild-type (WT) Smc5/6 was stimulated by dsDNA.

c) Effects of Smc5/6^{KE} and Smc5/6^{EQ} on the SUMOylation of Smc5 and Smc6 without DNA in lower salt conditions. SUMOylation reactions were conducted as those in Figure 5a except that the buffer contained 40 mM KCl. A representative result is shown (left) and quantification of at least three independent reactions per condition is plotted (right) as in Figure 5b, with means, SDs, and p-values calculated from Welch's t-test indicated (ns, not significant; n=6 for WT; n=3 for each mutant complex).

Supplementary Fig. 6. Multiple non-E3 subunits of Smc5/6 are required for *in vivo* Nse2-mediated SUMOylation.

a-b) Detection of Smc5 and Smc6 SUMOylation in cell extracts. His-Flag-tagged yeast SUMO (HF-Smt3) led to retardation of the migration of SUMOylated Smc5 (Su-Smc5) or Smc6 (Su-Smc6). Strains containing untagged Smc5 and Smc6 were used as controls.

c) Acute loss of Smc6, Nse4, or Nse6 reduces Smc5 SUMOylation levels in cells. Degraded subunits of Smc5/6 were C-terminally tagged with the tandem AID degron and the Flag epitope. All strains contained the TIR protein to allow AID-fusion protein degradation upon adding IAA[58,63]. Immunoblots monitoring subunit degradation were probed with the anti-Flag antibody (top). All strains also contained TAP-tagged Smc5 to assess its SUMOylation, which was detected by immunoblotting using an anti-TAP antibody (mid-bottom). Long exposure reveals both unmodified Smc5 and SUMOylated form of Smc5.

d) Acute loss of Smc5, Nse4, or Nse6 reduces Smc6 SUMOylation. Experiments were conducted and results are shown as in panel c.

e) Schematic to illustrate enrichment of SUMOylated proteins, including Myc-tagged Sgs1, on Ni-NTA resin from cells containing His8-tagged SUMO and subsequent detection of SUMOylated form of Sgs1 on immunoblots probed with an anti-Myc antibody.

f) SUMOylated forms of Sgs1-Myc (Su-Sgs1). Immunoblots probed with an anti-Myc antibody showed that Sgs1 SUMOylation was only defected when cells contain His8-tagged Smt3 (lane 3). Cells were treated with genotoxin MMS to induce Sgs1 SUMOylation. Protein loading is shown by Ponceau stain (stain).

g) Acute loss of Smc5, Nse3, or Nse5 reduces the level of Sgs1 SUMOylation. Cells were treated and results are shown as in panel f.

Supplementary Fig. 7. Mutating the ATP binding or hydrolysis site on Smc5 leads to cell lethality

a-c) Mutating the key residue of Smc5 involved in ATP binding or hydrolysis results in lethality. Diploid yeast strains with indicated genotypes were examined by tetrad analyses. Three representative tetrads are shown for each diploid strain and are numbered. While HA tagged wild-type Smc5 supported cell growth (**a**), HA-tagged smc5^{KE} (mutated for ATP binding site) (**b**) or HA-tagged smc5^{EQ} (mutated for ATP hydrolysis site) (**c**) failed to support cell growth.

Supplementary Fig. 8. Structural comparison of Nse2 bound to Smc5 versus bound to Smc5-Smc6.

a) The structure of Nse2 bound to a portion of Smc5's coiled coil arm region, the Ubc9 E2 and a SUMO moiety bound to the backside of Ubc9. (**Top**) a schematic of the Nse2 domains mapped in the structure (PDB:7P47), with the dashed line indicating an internal deletion in this shortened version of Nse2[33].The Nse2 C-terminal SUMO interaction motif (SIM) and its SP-RING SUMO E3 domain are indicated. (**Bottom**) Nse2 is depicted in ribbon representation, whereas the Smc5-arm region, Ubc9, and the SUMO moiety bound to the backside of Ubc9 are depicted in surface representations. While the SP-RING domain of Nse2 contacts Ubc9, its C-terminal SIM contacts the SUMO molecule located to the backside of Ubc9. The SUMO conjugated to the active site of Ubc9 is omitted for clarity.

b) The structure of Nse2 bound to the Smc5-Smc6 arm regions as seen in the ATP-free holo-Smc5/6 structure. (**Top**) a schematic of the Nse2 domains mapped in the indicated structure (PDB: 7YQH), with its wedge domain (H15 to L30) that interacts with the Smc6 coiled coil arm region and the SP-RING E3 domain indicated[23]. Not highlighted here is a large portion of the Nse2 N-terminal region bound to the Smc5 coiled coil arm region. (**Bottom**) Nse2 is depicted in ribbon representation, with its SP-RING E3 domain (black square) indicated. Nse2-bound arm regions of Smc5 and Smc6 that are associated with each other in the ATP-free I-shaped conformation are shown in surface representations. The boundary residues of the Nse2 wedge region engaged in Smc6 binding are marked.

c) Superimposition of the Nse2 structures engaged only with the Smc5 arm region and engaged with the arm regions of Smc5 and Smc6. The Nse2 structures shown in panel a and b were superimposed through alignment of the Nse2 region covering 140 to 237 amino acids. The two structures align well throughout the Nse2 protein, with a noticeable exception at the C-terminal SIM region of protein, which is indicated by the red arrow.

d) Superimposition of Nse2 structures in the context of its binding partners. (**Left**) structures shown in panel a and b are superimposed in the same manner as in panel c. (**Right**) an enlarged view of the interaction between the Nse2 C-terminal SIM and the Smc5 arm region as seen in the I-shaped holo-Smc5/6 (top). A detailed view of the interaction between the Nse2 C-terminal SIM with the SUMO moiety bound to the backside of the Ubc9 SUMO E2 is shown (bottom).

Supplementary Table 1. Yeast strains used in this study.

Strain	Genotype	Source	Figures
T1107-5D	*Smc5-AID-3Flag::KAN*	(Peng et al., 2018)	6a
T2252-18-5B	*Smc5-DNAm (K89, 97-98, 145-147, 192 all to A) -AID-3Flag::KAN*	(Yu et al., 2022)	6a
X9209-3B	*Smc5-6HA::NAT*	This study	6b, S6a
X9298-3C	*Smc5-6HA::NAT nse4-DNAm (R251, R256-258 all to E)::NAT*	This study	6b
X9210-4C	*Smc6-6HA::NAT*	This study	6c, S6b
X9248-10A	*Smc6-6HA::NAT smc5-DNAm (K89, 97-98, 145-147, 192 all to A)::NAT*	This study	6c
X9373-2	*Smc6-6HA::NAT/+ nse4-DNAm (R251, R256-258 all to E)::NAT /+*	This study	6d
X6684-8A	*8His-Smt3::TRP1 Sgs1-9myc::KAN*	This study	6e, S6f
X9256-10A	*8His-Smt3::TRP1 Sgs1-9myc::KAN smc5-DNAm (K89, 97-98, 145-147, 192 all to A)::NAT*	This study	6e
X9482-13A	*8His-Smt3::TRP1 Sgs1-9myc::KAN nse4-DNAm (R251, R256-258 all to E)::NAT*	This study	6e
T2402-21	*Smc5-4GS-IAA(111)-3flag::HIS3/Smc5-6HA::NAT ura3-1::ADH1-OsTIR1-9Myc::URA3/ura3-1*	This study	7a-7d, S7a
T2402-9	*Smc5-4GS-IAA(111)-3flag::HIS3/smc5-K75E-6HA::NAT ura3-1::ADH1-OsTIR1-9Myc::URA3/ura3-1*	This study	7a-b, S7b
T2403-1	*Smc5-4GS-IAA(111)-3flag::HIS3/smc5-E1015Q-6HA::NAT ura3-1::ADH1-OsTIR1-9Myc::URA3/ura3-1*	This study	7c-d, S7c
T2426-22	*Smc5-4GS-IAA(111)-3flag::HIS3/Smc5-6HA::NAT ura3-1::ADH1-OsTIR1-9Myc::URA3/ura3-1 Sgs1-9myc::KAN/+ 8HIS-Smt3::TRP/+*	This study	7e
T2426-19	*Smc5-4GS-IAA(111)-3flag::HIS3/smc5-K75E-6HA::NAT ura3-1::ADH1-OsTIR1-9Myc::URA3/ura3-1 Sgs1-9myc::KAN/+ 8HIS-Smt3::TRP/+*	This study	7e
T2427-24	*Smc5-4GS-IAA(111)-3flag::HIS3/smc5-E1015Q-6HA::NAT ura3-1::ADH1-OsTIR1-9Myc::URA3/ura3-1 Sgs1-9myc::KAN/+ 8HIS-Smt3::TRP/+*	This study	7e
X3579-11d	*HF-Smt3::LEU2*	(Dhingra et al., 2019)	S6a-b
X9333-4C	*HF-Smt3::LEU2 Smc5-6HA::NAT*	This study	S6a
X9248-2A	*HF-Smt3::LEU2 Smc6-6HA::NAT*	This study	S6b
X4410-18D	*Smc6-4GS-IAA(111)-3flag::HIS3 ura3-1::ADH1-OsTIR1-9Myc::URA3 Smc5-TAP::TRP1*	(Peng et al., 2018)	S6c
X4413-11A	*Nse4-AID111-3FLAG::KANMX ura3-1::ADH1-OsTIR1-9Myc::URA3 Smc5-TAP::KAN*	(Peng et al., 2018)	S6c
X4461-6B	*Nse6-AID111-FLAG::HIS3 ura3-1::ADH1-OsTIR1-9Myc::URA3 Smc5-TAF::KAN*	(Peng et al., 2018)	S6c
X4463-2A	*Smc5-4GS-IAA(111)-3flag::HIS3 ura3-1::ADH1-OsTIR1-9Myc::URA3 Smc6-TAP::URA3*	This study	S6d
X4414-16D	*Nse4-AID111-3FLAG::KANMX ura3-1::ADH1-OsTIR1-9Myc::URA3 Smc6-TAP::URA*	This study	S6d
X4611-14A	*Nse6-AID111-FLAG::HIS3 ura3-1::ADH1-OsTIR1-9Myc::URA3 Smc6-TAP::URA*	This study	S6d
X8747-19B	*8His-Smt3::TRP1*	This study	S6f
X6684-2B	*Sgs1-9myc::KAN*	This study	S6f

X9596-5D	*Smc5-4GS-IAA(111)-3flag::HIS3 ura3-1::ADH1-OsTIR1-9Myc::URA3 Sgs1-9myc::KAN 8HIS-Smt3::TRP*	This study	S6g
X9594-3D	*Nse3-4GS-IAA(111)-3flag::HIS3 ura3-1::ADH1-OsTIR1-9Myc::URA3 Sgs1-9myc::KAN 8HIS-Smt3::TRP*	This study	S6g
X9595-6B	*Nse5-4GS-IAA(111)-3flag::HIS3 ura3-1::ADH1-OsTIR1-9Myc::URA3 Sgs1-9myc::KAN 8HIS-Smt3::TRP*	This study	S6g

Supplementary Table 2. Plasmids used in this study.

**Name**	**Vector information**	**Source**
P675	pET-Smc5/6-6mer	(Taschner et al., 2021)
pXZ921	pET-Smc5/6-6mer-Smc5^{DNAm}	This study
pXZ922	pET-Smc5/6-6mer-Smc6^{DNAm}	(Chang et al., 2026)
pXZ919	pET-Smc5/6-6mer-Nse3^{DNAm}	This study
pXZ920	pET-Smc5/6-6mer-Nse4^{DNAm}	This study
P676	pET-Smc5/6-6mer-EQ	(Taschner et al., 2021)
pXZ925	pET-Smc5/6-6mer-KE	This study
	pFastBac-HTB-Flag-Sgs1	(Niu et al., 2010)
pLK79	pET11c-V5-Top3	(Niu et al., 2010)
	pGEX-6P-Rmi1	(Niu et al., 2010)
pXZ114	pET15b-Mms21	(Duan et al., 2009)
pXZ115	pET28a-Smc5	(Duan et al., 2009)
pXZ998	pRSFDuet-GST-Aos1	(Zhao & Blobel, 2005)
pXZ999	pET22-Uba2	(Zhao & Blobel, 2005)
p541	pET21a-Smt3	lab collection
G1827	pET-Ubc9	lab collection

Supplementary Table 3. DNA Substrates used in this study.

DNA	Oligo	Sequence
HJ	H3 (80nt)	5’-TTGATAAGAGGTCATTTGAATTCATGGCTTAGAGCTTAATTGCTGAATCTGGTG CTGGGATCCAACATGTTTTAAATATG-3’
	H5 (80nt)	5’-CATATTTAAAACATGTTGGATCCCAGCACCAGATTCAGCATACGTTACCGATCG TACGTTCGATGCTGGCTACTGCTAGC-3’
	H7 (80nt)	5’-GCTAGCAGTAGCCAGCATCGAACGTACGATCGGTAACGTAGTCGATTATCGA GATCAAGCTAGCATAGCCATAGCGCGAC-3’
	H8 (80nt)	5’-GTCGCGCTATGGCTATGCTAGCTTGATCTCGATAATCGACATTAAGCTCTAAG CCATGAATTCAAATGACCTCTTATCAA-3’
dsDNA	H3 (80nt)	5’-TTGATAAGAGGTCATTTGAATTCATGGCTTAGAGCTTAATTGCTGAATCTGGTG CTGGGATCCAACATGTTTTAAATATG-3’
	H4 (80nt)	5’- CATATTTAAAACATGTTGGATCCCAGCACCAGATTCAGCAATTAAGCTCTAAGC CATGAATTCAAATGACCTCTTATCAA-3’
	DS1 (30nt)	5’-GGACATCTTTGCCCACCTGCAGGTTCACCC-3’
	DS3 (30nt)	5’-GGGTGAACCTGCAGGTGGGCAAAGATGTCC-3’
	H1 (40nt)	5’-ATTAAGCTCTAAGCCATGAATTCAAATGACCTCTTATCAA-3’
	H9 (40nt)	5’-TTGATAAGAGGTCATTTGAATTCATGGCTTAGAGCTTAAT-3’
	H10 (50nt)	5’- TTGATAAGAGGTCATTTGAATTCATGGCTTAGAGCTTAATTGCTGAATCT-3’
	H11 (50nt)	5’- AGATTCAGCAATTAAGCTCTAAGCCATGAATTCAAATGACCTCTTATCAA-3’
	XX1 (60nt)	5’- ACGCTGCCGAATTCTACCAGTGCCTTGCTAGGACATCTTTGCCCACCTGCAG GTTCACCC-3’
	XX2C (60nt)	5’- GGGTGAACCTGCAGGTGGGCAAAGATGTCCTAGCAAGGCACTGGTAGAATTC GGCAGCGT-3’
	A2’ (90nt)	5’- GTCACTTGATAAGAGGTCATTTGAATTCATGGCTTAGAGCTTAATTGCTGAATC TGGTGCTGGGATCCAACATGTTTTAAATATGCAATG-3’
	A7 (90nt)	5’- CATTGCATATTTAAAACATGTTGGATCCCAGCACCAGATTCAGCAATTAAGCTC TAAGCCATGAATTCAAATGACCTCTTATCAAGTGAC-3’
ssDNA	H3 (80nt)	5’-TTGATAAGAGGTCATTTGAATTCATGGCTTAGAG CTTAATTGCTGAATCTGGTGCTGGGATCCAACATGTTTTAAATATG-3’
ss-dsDNA	H3 (80nt)	5’-TTGATAAGAGGTCATTTGAATTCATGGCTTAGAG CTTAATTGCTGAATCTGGTGCTGGGATCCAACATGTTTTAAATATG-3’
	H2 (40nt)	5’-ATTAAGCTCTAAGCCATGAATTCAAATGACCTCTT ATCAA-3’
